# Body trunk fat and insulin resistance in post-pubertal obese adolescents

**DOI:** 10.1590/S1516-31802008000200004

**Published:** 2008-03-06

**Authors:** Luana Caroline dos Santos, Isa de Pádua Cintra, Mauro Fisberg, Lígia Araújo Martini

**Keywords:** Adolescent, Body composition, Abdominal fat, Insulin resistance, Obesity, Adolescente, Composição corporal, Gordura abdominal, Resistência à insulina, Obesidade

## Abstract

**CONTEXT AND OBJECTIVE:**

Insulin resistance is a metabolic disorder commonly associated with excess body fat accumulation that may increase chronic disease risk. The present study was undertaken to evaluate the relationship between body composition and insulin resistance among obese adolescents.

**DESIGN AND SETTING:**

Cross-sectional study, at the Adolescence Center, Pediatric Department, Universidade Federal de São Paulo.

**METHODS:**

Body composition was assessed using dual-energy X-ray absorptiometry. Dietary intake was evaluated using a three-day dietary record. The biochemical evaluation comprised glucose, insulin, serum lipid, leptin and ghrelin measurements. Insulin resistance was calculated by means of the homeostasis model assessment of insulin resistance (HOMA-IR).

**RESULTS:**

Forty-nine post-pubertal obese adolescents participated in the study: 12 boys and 37 girls of mean age 16.6 (1.4) years and mean body mass index (BMI) of 35.0 (3.9) kg/m^2^. The mean glucose, insulin and HOMA values were 90.3 (6.4) mg/dl, 16.6 (8.1) μIU/ml and 3.7 (1.9), respectively. Hyperinsulinemia and insulin resistance were observed in 40.2% and 57.1% of the subjects, respectively. Adolescents with insulin resistance had higher BMI and body trunk fat. There was a trend towards higher leptin concentration in obese individuals with insulin resistance. Insulin resistance was positively correlated with body trunk fat, BMI, body fat mass (kg), leptin and body fat percentage. Furthermore, there was a negative correlation between HOMA-IR and lean body mass. The body composition predicted 30% of the HOMA-IR levels, according to linear regression models.

**CONCLUSION:**

Body trunk fat was significantly associated with insulin resistance, demonstrating the clinical importance of abdominal obesity during adolescence.

## RESUMO

**CONTEXTO E OBJETIVO:**

A resistência à insulina é uma desordem metabólica, comumente associada ao acúmulo excessivo de gordura corporal, que pode aumentar o risco de doenças crônicas. O objetivo foi avaliar a relação entre a composição corporal e a resistência à insulina em adolescentes obesos.

**TIPO DE ESTUDO E LOCAL:**

Estudo transversal, realizado no Centro de Atendimento e Apoio ao Adolescente, Departamento de Pediatria, Universidade Federal de São Paulo.

**MÉTODOS:**

Realizou-se avaliação da composição corporal, avaliação do consumo alimentar e avaliação bioquímica (dosagem de glicose, insulina, colesterol total e frações, leptina e grelina). A resistência à insulina (RI) foi calculada pelo HOMA-IR (Homeostasis Model Assessment of Insulin Resistance).

**RESULTADOS:**

49 adolescentes obesos [12 meninos e 37 meninas; idade média de 16.6 (1.4) anos; média de índice de massa corpórea (IMC) 35.0 (3.9) kg/m^2^] participaram do estudo. A concentração média de glicose, insulina e HOMA-IR foram de 90.3 (6.4) mg/dl, 16.6 (8.1) μUI/ml e 3.7 (1.9), respectivamente. Hiperinsulinemia e RI foram observadas em 40,2% e 57,1% dos adolescentes, meninos e meninas, respectivamente. Adolescentes com RI apresentaram maior IMC e gordura do tronco. A concentração de leptina apresentou tendência a ser maior nestes indivíduos. A RI foi positivamente correlacionada com a gordura do tronco, IMC, massa adiposa (kg), leptina e percentual de gordura corporal. Além disso, houve correlação negativa entre HOMA-IR com a massa magra. A composição corporal foi capaz de predizer 30% do HOMA-IR segundo os modelos de regressão linear.

**CONCLUSÃO:**

A gordura do tronco foi significativamente associada com a resistência à insulina, demonstrando a importância clínica da obesidade abdominal durante a adolescência.

## INTRODUCTION

Insulin resistance consists of impairment of the ability of insulin to control hepatic glucose production and enhance glucose clearance in target tissues. It is commonly associated with excess accumulation of body fat.^1^ Investigation of body fat distribution has demonstrated that visceral abdominal fat is strongly associated with insulin resistance in adults; however, the data on obese adolescents are limited.^2^ Decreased insulin sensitivity is the greatest risk factor for the development of type 2 diabetes and perhaps the greatest current health threat to children and adolescents.^3^

Insulin resistance also leads to the impairment of other biological actions of insulin, including its effects on lipid and protein metabolism, vascular endothelial function and gene expression. The requirement for insulin increases as cells become more resistant. The body can overcome this by secreting more insulin from the pancreatic beta cells, thereby inducing progressive loss of beta cell function, secondary to exhaustion of their secretory capacity. This combination of insulin resistance and beta cell dysfunction characterizes type 2 diabetes mellitus.^4,5^

The primary cause of type 2 diabetes mellitus in children and adolescents is excess weight at this stage of life.^2^ The prevalence of childhood obesity has more than doubled over the past 15 years in many regions of the world, including Brazil.^6^ Cintra et al. found an obesity rate of almost 10% among 8,020 adolescents aged 10 to 15 years, in an epidemiological study in the city of São Paulo.^7^

Metabolic complications of obesity such as dyslipidemia, increased blood pressure and hormonal disorders have been demonstrated in adolescents,^2^ and may be linked to body fat distribution, which in turn is heavily influenced by gender.

Adipose tissue accumulates in two main sites: abdominal and peripheral. In males, fat typically accumulates in the upper segment of the body (abdominal region). In females, adipose tissue accumulates particularly over the thighs in a pear-shaped gluteal distribution. Gender-related patterns of body fat deposition become established during puberty and, as with total body fat, show significant familial associations.^5,8^

As in adulthood, childhood obesity may lead to increased concentrations of leptin and decreased ghrelin. Leptin is thought to act as a marker for adiposity levels, mechanisms controlling dietary intake and, possibly, energy expenditure.^9^ Leptin levels have also been associated with insulin levels in different studies.^1,10^ The effects of ghrelin on energy homeostasis are opposite to those of leptin and its relationship with insulin has not been fully defined.^9^

## OBJECTIVE

Considering that insulin sensitivity and decreased insulin response are major pathophysiological components of obesity and type 2 diabetes,^3,8^ the present study was undertaken in order to evaluate the relationship of body composition to insulin resistance among adolescents.

## MATERIAL AND METHODS

### Study design

This cross-sectional study included adolescents recruited through community service agencies and newspaper advertisements. Subjects of both sexes were eligible if they were post-pubertal according to Tanner stages,^11^ were sedentary (with less than three hours of physical activity/week)^12^ and had a body mass index (BMI; the weight in kilograms divided by the square of the height in meters) that exceeded the 95^th^ percentile for their age and sex.^13^

The exclusion criteria were the known presence of chronic disease other then obesity (n = 4), use of medication that alters weight, glucose or lipid metabolism, weight greater than 120 kg (thereby making it impossible to measure body composition) (n = 6), prepubertal stage^11^ (n = 1), BMI under 95^th^ percentile for age and sex (n = 2).^13^

This study was performed at the Outpatient Clinics for Adolescents (CSCA) of Universidade Federal de São Paulo (Unifesp). CSCA is a public service for disease prevention and health promotion among adolescents in the city of São Paulo, Brazil.

The study was approved by the Ethics Committee of Universidade de São Paulo (USP) and by the Ethics Committee of Universidade Federal de São Paulo (Unifesp).

### Body composition

The body composition was assessed by means of dual-energy X-ray absorptiometry (DXA), using Hologic QDR 4500ª apparatus (Hologic Inc., Waltham, Massachusetts, United States). DXA results are divided into bone mass and soft tissue mass. Soft tissue mass is then divided into fat mass (trunk and peripheral) and lean body mass.

### Dietary assessment

All subjects were instructed to write down their total daily food intake for three non-consecutive days, using household measurements and describing the amount of each food consumed. The records were received and evaluated by a trained nutritionist. Nutrient intakes were calculated using the NutWin 1.5 software (2002), which is nutritional software developed by Unifesp (São Paulo, Brazil).

### Biochemical assessment

A venous blood sample was taken after 12 hours of fasting, to measure biochemical parameters. Serum glucose concentrations were determined using an ultraviolet spectrophotometer (model 1601PC, Shimadzu Corp., Kyoto, Japan). Serum insulin levels were determined using a radioimmunoassay kit (Molecular Research Center, Inc., Cincinnati, Ohio, United States). The reference values adopted were from the Expert Committee on the Diagnosis and Classification of Diabetes Mellitus.^14^

Insulin resistance was estimated using the homeostasis model assessment of insulin resistance (HOMA-IR).^15^ Any adolescent with a HOMA-IR value higher than 3.16 was considered insulin-resistant.^3^

Triglycerides, total cholesterol and high-density lipoprotein cholesterol (HDL-c) were evaluated by means of enzymatic colorimetric methods. Low-density lipoprotein cholesterol (LDL-c) was calculated using the Friedewald equation.

Leptin and ghrelin levels were determined by means of a radioimmunoassay procedure using a commercial kit (Linco Research, St Charles, Missouri, United States). The intra and inter-assay coefficients of variation were 3.7 – 7.5% and 5.3% for leptin and 3.2 – 8.9% and 13.6% for ghrelin, respectively.

Biochemical assessments were performed by a specialized laboratory within Unifesp.

### Statistical analysis

The results relating to continuous data are reported as means (with standard deviation). The Kolmogorov-Smirnov normality test was performed. Student's t test and Pearson's correlations were used to compare means and to verify associations between variables, respectively. Linear regression models were used to identify factors possibly related to HOMA-IR, which was considered to be the dependent variable. The anthropometric and body composition measurements, dietary intake data and biochemical parameter concentrations were the independent variables. All variables showing significant correlations with HOMA-IR were tested by means of the stepwise method. P-values < 0.05 were considered significant. The data were analyzed using the Statistical Package for the Social Sciences version 12.0 for Windows (SPSS Inc., Chicago, Illinois, United States).

## RESULTS

Forty-nine post-pubertal obese adolescents participated in the study: 12 boys and 37 girls of mean age 16.6 (1.4) years and mean BMI of 35.0 (3.9) kg/m^2^. As expected, the girls presented significantly greater total body fat, trunk fat and peripheral fat, and lower weight and height, in comparison with the boys (Table 1).

**Table 1.** Physical characteristics of post-pubertal obese adolescents, São Paulo, 2004

	Boys (n = 12)	Girls (n = 37)
Age (years)	16.1 (1.2)	16.8 (1.4)
Weight (kg)	106.7 (8.6)	92.9 (11.7)*
Height (m)	1.73 (0.6)	1.63 (0.4)*
Body mass index (kg/m^2^)	35.4 (3.3)	34.9 (4.2)
Total body fat (%)	35.1 (4.3)	42.8 (3.6)*
Body trunk fat (%)	35.1 (4.7)	42.7 (4.4)*
Body peripheral fat (%)	35.8 (4.8)	45.4 (4.8)*
Lean body mass (kg)	63.1 (3.4)	48.6 (4.9)*

* p < 0.001.

The mean glucose level was 90.3 (6.4) mg/dl (range: 76 - 110 mg/dl) and none of the adolescents showed glucose classified as diabetes mellitus (≥ 126 mg/dl). However, impaired glucose, as measured by fasting glucose greater than 100 mg/dl, was found in 5% of the adolescents.

The mean insulin concentration was 16.6 (8.1) uIU/ml and the mean HOMA-IR was 3.7 (1.9). Hyperinsulinemia and insulin resistance were observed in 40.2% and 57.1% of the adolescents, respectively. There was no statistical difference between genders, in relation to these parameters.

Compared with subjects without insulin resistance, those with insulin resistance had higher BMI and body trunk fat (Table 2). Leptin concentrations tended to be higher in obese subjects with insulin resistance. There were no statistical differences in dietary intake, in relation to insulin resistance status.

**Table 2.** Body composition, biochemical profile and dietary intakes according to insulin resistance (IR) status among post-pubertal obese adolescents, São Paulo, 2004

	Adolescents without IR (n = 21)	Adolescents with IR (n = 28)
Body mass index (kg/m^2^)	33.5 (3.2)	36.3 (4.0)*
Body fat (%)	40.0 (6.1)	41.6 (4.1)
Body trunk fat (%)	38.8 (5.9)	42.5 (4.7)†
Body peripheral fat (%)	43.1 (7.3)	43.1 (5.1)
Lean body mass (kg)	51.8 (9.0)	52.4 (6.8)
Total cholesterol (mg/dl)	162.3 (25.7)	159.5 (27.9)
LDL-cholesterol (mg/dl)	93.8 (20.6)	91.4 (24.8)
HDL-cholesterol (mg/dl)	50.1 (10.9)	46.3 (7.8)
Triacylglycerol (mg/dl)	91.6 (32.9)	107.3 (47.3)
Leptin (ng/dl)	36.2 (20.6)	46.5 (15.9)‡
Ghrelin (ng/dl)	4.5 (2.2)	4.7 (2.1)
Energy intake (kcal)	1953.8 (755.9)	1834.7 (838.2)
Carbohydrate (%)	51.8 (5.9)	52.5 (8.9)
Protein (%)	15.6 (4.3)	15.5 (5.3)
Lipids (%)	32.5 (4.9)	32.0 (8.4)

LDL = low-density lipoprotein; HDL = high-density lipoprotein;

* p < 0.01;

† p < 0.05;

‡ p = 0.07.

**Figure 1:**
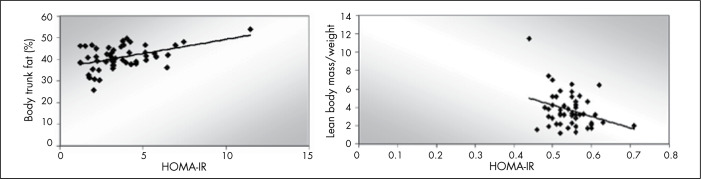
Correlation between homeostasis model assessment of insulin resistance (HOMA-IR) and body trunk fat and lean body mass among post-pubertal obese adolescents, São Paulo, 2004. A) Correlation between HOMA-IR and body trunk fat (y = 1.3153x + 36.029; r = 0.457, p = 0.001); B) Correlation between HOMA-IR and lean body mass (y = 12.442x + 10.455; r = −0.306, p = 0.03).

Insulin resistance was positively correlated with body trunk fat (r = 0.457; p = 0.001), BMI (r = 0.417; p = 0.003), body fat mass (kg) (r = 0.386; p = 0.006), leptin (r = 0.307; p = 0.045) and body fat percentage (r = 0.285; p = 0.047). Furthermore, there was a negative correlation between HOMA-IR and lean body mass (Figure 1).

As seen in Figure 1, one subject had higher HOMA-IR and body fat than seen among the other adolescents. The statistical analysis was performed without this subject. The correlation of insulin resistance with body trunk fat was seen to be independent of the presence or absence of this single outlier subject. However, the correlation of insulin resistance with lean body mass and body fat percentage was seen to disappear when the outlying subject was excluded.

Linear regression models were used in order to investigate the factors possibly associated with insulin resistance. Body composition predicted 30% of the HOMA-IR levels, and the best model for this was composed of body trunk fat and lean body mass, adjusted for age (Table 3). Other variables like BMI, body fat mass and leptin were tested, but they were not statistically significant in the linear model. The age was included in the final model as a control variable.

**Table 3.** Final linear model regression coefficients for factors associated with insulin resistance among post-pubertal obese adolescents, São Paulo, 2004

	B	p-value
β_0_	-31.983	-
Body trunk fat (%)	0.478	0.000
Lean mass	37.01	0.007
Age (years)	-0.239	0.173

Dependent variable: homeostasis model assessment of insulin resistance (HOMA-IR).

## DISCUSSION

The current study demonstrated a high association between body trunk fat and hyperinsulinemia and insulin resistance among obese adolescents. Although similar data were found by other investigators such as Freedman et al.,^16^ Yanovski et al.^17^ and Weiss et al.,^18^ this study is unique since none of the adolescents were diabetic, nor did they display other chronic diseases related to obesity.

Differently, Tershakovec et al. did not observe any significant correlation of insulin or HOMA-IR with visceral abdominal fat among obese children and adolescents. However, there are some differences between Tershakovec's study and ours. In their study, the adolescents were younger, with a mean age of 11.8 (0.5) years.^2^ Age is an important issue, since fat distribution may differ between the prepubertal and pubertal stages.^17^

The association between abdominal fat deposition and insulin resistance is not fully understood. However, higher rates of free fatty acid (FFA) and cytokine production have been implicated in this process.^5^

Different fat depots vary in their responsiveness to the hormones that regulate lipolysis, such that visceral depots are less responsive to the antilipolytic action of insulin.^5^ The resulting high rate of FFA turnover in visceral fat depots has important physiological consequences, because of the direct link between visceral depots and the liver through the portal vein. The delivery of FFAs into the portal circulation by visceral depots may lead to increased triglyceride and glucose synthesis and reduced hepatic clearance of insulin.^18^ Therefore, it has been hypothesized that the FFAs released from visceral adipose depots are important factors contributing to the relationship between visceral fat and reduced insulin.

In our study, dietary intake, biochemical parameters and ghrelin were not correlated with insulin resistance. However, leptin concentration was associated with HOMA values and higher leptin concentration was found among adolescents with insulin resistance.

Tershakovec et al. found that leptin levels were associated with insulin and HOMA-IR, independent of total fat and subcutaneous abdominal fat.^2^ This could be consistent with leptin resistance in individuals with high insulin concentrations, or it could be an adaptive mechanism to help prevent further weight gain.^19^

Roemmich et al. also found a positive association between serum concentrations of leptin and HOMA and fasting serum insulin concentrations, even after adjusting for total and regional adiposity and physical characteristics.^1^ This result confirms that E-adipose tissue and the pancreas are functionally connected through an “adipoinsulin axis”.^20^

Ghrelin modulates circulating glucose levels via growth hormone release, thus increasing insulin resistance, and stimulating gluconeogenesis.^21,22^ However, ghrelin levels are lower in cases of human obesity^23,24^ Ikezaki et al. found that fasting plasma ghrelin levels were negatively correlated with insulin resistance in 49 obese Japanese children (r = −0.317; p < 0.05).^24^ Similar results were found by Tschöp et al. among obese adult Caucasians and Pima Indians.^23^ In the present study, no association between ghrelin and insulin resistance was observed, maybe because of differences in age and pubertal stages in relation to other studies, and because of the lower sensitivity of this hormone to insulin change, in comparison with leptin. According to some authors, the effect of ghrelin on insulin secretion is controversial.^21,22,25^

Future studies to determine the control mechanisms involved in abdominal fat accumulation may provide novel forms of treatment for type 2 diabetes and other chronic disorders associated with visceral obesity. Genetic studies have already demonstrated that up to 50% of the variance in abdominal fat mass may be attributed to genetic factors, thus suggesting that development of therapies targeting visceral fat accumulation may not be beyond reach.^5^

Our study does have certain limitations. The small sample size could limit the power of the analyses. However, the similar results seen by other investigators support our findings.^14–16^ No functional measurements of insulin response were investigated, since such measurements would have required the use of invasive and expensive techniques such as clamps. Instead, HOMA-IR evaluation provides high sensitivity and specificity for measuring insulin resistance, especially among adolescents.^3^

Thus, the present study of body composition, regional fat distribution and metabolic factors among obese adolescents underscores the complexity of these relationships. Given the epidemic of pediatric obesity and the limited data relating to adolescents, especially obese adolescents, it is essential to gain a better understanding of the factors such as body composition and insulin resistance that are associated with this epidemic.

In any event, there is increasing evidence to support the observation that, by the time glucose tolerance or fasting glucose levels become impaired, appreciable ^β^-cell destruction may already have occurred. Thus, it seems likely that attempts to prevent type 2 diabetes will be more successful if intervention is begun while blood glucose levels are still within the normal range.

## CONCLUSION

The present study demonstrated high prevalence of insulin resistance and impaired glucose tolerance associated with body trunk fat, among obese non-diabetic adolescents. It indicates the need for an intervention program in early life in order to prevent diabetes and other metabolic complications due to obesity. Furthermore, improvements in dietary intake and physical activity are essential, especially for adolescents, in order to promote a better quality of life in the future.

## References

[1] Roemmich JN, Clark PA, Lusk M (2002). Pubertal alterations in growth and body composition. VI. Pubertal insulin resistance: relation to adiposity, body fat distribution and hormone release. Int J Obes Relat Metab Disord.

[2] Tershakovec AM, Kuppler KM, Zemel BS (2003). Body composition and metabolic factors in obese children and adolescents. Int J Obes Relat Metab Disord.

[3] Keskin M, Kurtoglu S, Kendirci M, Atabek E, Yazici C (2005). Homeostasis model assessment is more reliable than the fasting glucose/insulin ratio and quantitative insulin sensitivity check index for assessing insulin resistance among obese children and adolescents. Pediatrics.

[4] Sinaiko AR, Jacobs DR, Steinberger J (2001). Insulin resistance syndrome in childhood: associations of the euglycemic insulin clamp and fasting insulin with fatness and other risk factors. J Pediatr.

[5] Ali AT, Crowther NJ (2005). Body fat distribution and insulin resistance. S Afr Med J.

[6] Wang Y, Monteiro C, Popkin BM (2002). Trends of obesity and underweight in older children and adolescents in the United States, Brazil, China, and Russia. Am J Clin Nutr.

[7] Cintra Ide P, Passos MA, Fisberg M, Machado HC (2007). Evolução em duas séries históricas do índice de massa corporal em adolescentes. [Evolution of body mass index in two historical series of adolescents]. J Pediatr (Rio J).

[8] Slyper AH (1998). Childhood obesity, adipose tissue distribution, and the pediatric practitioner. Pediatrics.

[9] Vendrell J, Broch M, Vilarrasa N (2004). Resistin, adiponectin, ghrelin, leptin, and proinflammatory cytokines: relationships in obesity. Obes Res.

[10] Huang KC, Lin RC, Kormas N (2004). Plasma leptin is associated with insulin resistance independent of age, body mass index, fat mass, lipids, and pubertal development in nondiabetic adolescents. Int J Obes Relat Metab Disord.

[11] Tanner JM (1962). Growth at adolescence.

[12] Institute of Medicine (2002). Dietary reference intakes for energy, carbohydrate, fiber, fat, fatty acids, cholesterol, protein, and amino acids (macronutrients).

[13] Centers for Disease Control and Prevention National Center for Health Statistics.

[14] Expert Committee on the Diagnosis and Classification of Diabetes Mellitus (2003). Report of the expert committee on the diagnosis and classification of diabetes mellitus. Diabetes Care.

[15] Matthews DR, Hosker JP, Rudenski AS, Naylor BA, Treacher DF, Turner RC (1985). Homeostasis model assessment: insulin resistance and beta-cell function from fasting plasma glucose and insulin concentrations in man. Diabetologia.

[16] Freedman DS, Dietz WH, Srinivasan SR, Berenson GS (1999). The relation of overweight to cardiovascular risk factors among children and adolescents: the Bogalusa Heart Study. Pediatrics.

[17] Yanovski JA, Yanovski SZ, Cutler GB, Chrousos GP, Filmer KM (1996). Differences in the hypothalamic-pituitary-adrenal axis of black girls and white girls. J Pediatr.

[18] Weiss R, Dziura J, Burgert TS (2004). Obesity and the metabolic syndrome in children and adolescents. N Engl J Med.

[19] Spear BA (2002). Adolescent growth and development. J Am Diet Assoc.

[20] Björntorp P (1991). Metabolic implications of body fat distribution. Diabetes Care.

[21] Yildiz BO, Suchard MA, Wong ML, McCann SM, Licinio J (2004). Alterations in the dynamics of circulating ghrelin, adiponectin, and leptin in human obesity. Proc Natl Acad Sci U S A.

[22] McAuley KA, Williams SM, Mann JI (2001). Diagnosing insulin resistance in the general population. Diabetes Care.

[23] Tschöp M, Weyer C, Tataranni PA, Devanarayan V, Ravussin E, Heiman ML (2001). Circulating ghrelin levels are decreased in human obesity. Diabetes.

[24] Ikezaki A, Hosoda H, Ito K (2002). Fasting plasma ghrelin levels are negatively correlated with insulin resistance and PAI-1, but not with leptin, in obese children and adolescents. Diabetes.

[25] Ueno H, Yamaguchi H, Kangawa K, Nakazato M (2005). Ghrelin: a gastric peptide that regulates food intake and energy homeostasis. Regul Pept.

